# Circular RNA CircZNF644 Facilitates Circulating Follicular Helper T Cells Response in Patients with Graves' Disease

**DOI:** 10.1155/2024/9527268

**Published:** 2024-06-27

**Authors:** Yingzhao Liu, Xuehua Wang, Juan Xu, Qian Xu, Jie Xing, Junli Zou, Shengjun Wang, Huiyong Peng

**Affiliations:** ^1^ Department of Endocrinology The Affiliated People's Hospital of Jiangsu University Zhenjiang Medical School of Nanjing Medical University, Zhenjiang 212002, China; ^2^ Department of Endocrinology The Fourth Affiliated Hospital of Jiangsu University, Zhenjiang 212001, China; ^3^ Department of Critical Care Medicine The Affiliated People's Hospital of Jiangsu University Zhenjiang Medical School of Nanjing Medical University, Zhenjiang 212002, China; ^4^ Department of Laboratory Medicine The Affiliated People's Hospital of Jiangsu University Zhenjiang Medical School of Nanjing Medical University, Zhenjiang 212002, China; ^5^ Department of Genetic Toxicology The Key Laboratory of Modern Toxicology of Ministry of Education Center for Global Health School of Public Health Nanjing Medical University, Nanjing 211100, China

## Abstract

Aberrant accumulation of circulating follicular helper T cells (cTfh) has been found in the peripheral blood mononuclear cells (PBMCs) of Graves' disease (GD) patients. However, the underlying mechanism that contributes to the imbalance of cTfh cells remains unknown. Previously, studies described a GD-related circular RNAs (circRNAs)-circZNF644 that might be associated with cTfh cells. This study aimed to investigate the role of circZNF644 on cTfh cells in GD patients. Here, we found that circZNF644 was highly stable expression in the PBMCs of GD patients, which was positively correlated with the serum levels of TSH receptor autoantibodies (TRAb). Knockdown of circZNF644 caused a reduction of the proportion of cTfh cells in vitro. Mechanistically, circZNF644 served as a ceRNA for miR-29a-3p to promote ICOS expression, resulting in increased cTfh cells. In the PBMCs of GD patients, circZNF644 expression was positively correlated with ICOS expression and the percentage of cTfh cells, but negatively related to miR-29a-3p expression. Additionally, a strong relationship between circZNF644 and IL-21 was revealed in GD patients, and silencing of circZNF644 inhibited IL-21 expression. Our study elucidated that elevated expression of circZNF644 is a key feature in the development of GD and may contribute to the pathogenic role of cTfh cells in GD.

## 1. Introduction

Graves' disease (GD) is a chronic inflammatory autoimmune thyroid disorder characterized by diffuse goiter, hyperthyroidism, palpitation (tachycardia), weight loss, heat intolerance, and, in approximately 25% of patients, Graves ophthalmopathy (GO) [1, 2]. It affects millions worldwide with an annual incidence of 20–40 cases/100,000 individuals globally [3]. According to the present data, GD can occur at all ages, predominantly in 30–60 years old, and it is more common in females than in males (5–10 times) [4]. The pathophysiology of GD is caused by the presence of antithyroid stimulating hormone (TSH)-receptor antibodies (TRAb) to stimulate TSH receptor on thyroid follicular cells, eventually leading to elevated thyroid hormone levels and low TSH levels [5]. However, the exact aetiology of GD has not yet been fully elucidated. Although the occurrence and development of GD is the result of multiple risk factors [3], the leading cause is the infiltration of extensive immune cells causing an abnormal immune response. Studies over the past few years have highlighted the importance of T follicular helper (Tfh) cells in the pathogenesis of GD [6, 7, 8], which have been shown to help B cells produce antibodies in the face of antigenic challenge [9, 10].

Tfh cell lineage is a specialized subset of CD4^+^ T helper cells that play a pivotal role in orchestrating the humoral arm of adaptive immune responses [11]. The prominent role is homing to the B cell follicle for repeated interaction with B cells by persistent expression of C-X-chemokine receptor 5 (CXCR5) [12]. Such Tfh cells also bear a canonical characteristic phenotype including CD40 ligand (CD40L), inducible T cell costimulator (ICOS), programmed death-1 (PD-1), and B and T lymphocyte attenuator (BTLA) [13]. In addition to the classical germinal center (GC) Tfh cells in secondary lymphoid organs, it has been increasingly appreciated that there is a small subset of analogous cells in human peripheral blood with “Tfh-like” characteristics, frequently termed as circulating Tfh (cTfh) cells [14]. Curiously, such Tfh-like cells share functional properties with Tfh cells but exhibit a greater capacity for IL-21 production and superior B cell helper capacity [15]. Numerous studies have indicated that patients with autoimmune diseases have increased proportion of cTfh cells in peripheral blood [11], and our previous data also showed an increased frequency of cTfh cells in GD, which are closely associated with disease activity [7]. Therefore, effective regulation of cTfh cells to maintain stability of the immune microenvironment is a possible strategy for the prevention and treatment of GD.

Recently, circular RNAs (circRNAs) are extensively studied for their unique roles in biological processes as well as in development and progression of human diseases [16, 17, 18]. CircRNAs are a group of endogenous biomolecules characterized by covalently closed cyclic structure lacking poly-adenylated tails in eukaryotes [19]. The tissue-specific and cell-specific expression patterns of circRNAs impart their distinctive biological functions, including miRNA or protein inhibitors (“sponge”), transcriptional regulators, and protein templates [16]. Unlike their canonically spliced linear RNAs, circRNAs have long half-lives and are resistant to Ribonuclease R (RNase R) degradation in cells [20]. Recently, there has been an explosion in the number of studies about circRNAs in immune regulation and immune diseases, owing to the development of biochemical enrichment strategies and computational algorithms. However, the existing research on circRNAs and GD is quite limited. In our previous study, a GD-related circRNA, hsa_circ_0114427, was high-expressed in the peripheral blood mononuclear cells (PBMCs) by using next-generation sequencing (NGS) [21]. Intriguingly, miR-29a-3p was predicted to potentially bind to the hsa_circ_0114427 sequence through bioinformatics. Meanwhile, our previous data identified a notable downregulated miR-29a-3p that affects ICOS expression in GD patients [22]. Herein, it has come into the spotlight with the potential relationship between hsa_circ_0114427 and cTfh cells in GD.

In this study, we first verified circZNF644 expression in GD patients. Then, we investigated the role and mechanism of circZNF644 contributing to cTfh cells by a series of functional and molecular experiments. Our study provides new insights into understanding the mechanism of cTfh cells imbalance in GD.

## 2. Materials and Methods

### 2.1. Subjects and Samples

A study was conducted involving 36 GD patients who visited the outpatient clinic of endocrinology at the First People's Hospital of Zhenjiang, the key station between August 2020 and February 2021. GD was diagnosed which based on the criteria proposed by the Thyroid Group of Chinese Society of Endocrinology of Chinese Medical Association. Patients who were combined with malignancy, allergy, hematological diseases, other thyroid and autoimmune diseases, or pregnant, lactating, or taken immunotherapy drugs within 3 months prior to this study were excluded from the study. Among them, eight GD patients had received previous treatment for GD and the indicators of thyroid function returned to normal range. The therapeutic schemes were as follows: (i) 20–30 mg/day of methimazole for the first phase and the dose was reduced to 5–15 mg when patients achieved the remission stage; (ii) 300–500 mg/day of propylthiouracil therapy for the first phase and 25–100 mg/day for maintaining when patients achieved the remission stage. Thirty-eight sex- and age-matched healthy adult volunteers were included as the controls. Thyroid disease, tumors, infectious diseases, autoimmune diseases as well as abnormal serological features were excluded from the study. The main clinical characteristics of GD patients and controls are summarized in Table 1.

All the sampling procedures of the study were approved by the ethics committee of the First People's Hospital of Zhenjiang and the ethics approval number was K-20200012-Y. The fresh blood samples were collected from the subjects at the First People's Hospital of Zhenjiang after written informed consent was obtained from the subjects. All operations in this study adhered to standard biosecurity and institutional safety procedures.

### 2.2. Laboratory Measurements

The serum levels of free triiodothyronine (FT3), free thyroxine (FT4), TSH, anti-thyroglobulin antibody (TgAb), and anti-thyroid peroxidase autoantibody (TPOAb) were measured by CLIA method using LDX-800 system (Beckman Coulter, California, USA). We measured the serum levels of TRAb by ECLIA method using Cobas 6000 system (Roche, Basel, Switzerland).

### 2.3. Cell Isolation and Culture In Vitro

Human fresh blood samples were collected from the volunteers, and then separated by density-gradient centrifugation over Ficoll-Hypaque solution (Haoyang Biological Technology Co., Tianjin, China) into PBMCs for subsequent experiments. The isolated PBMCs were divided into two parts, one of which was stored at −80°C for quantitative real-time polymerase chain reaction (qRT-PCR), and the other one was cultured with RPMI-1640 medium (Gibco, California, USA) containing 10% fetal bovine serum (Gibco) 1640 for flow cytometry (FCM). HEK293T cells were cultured in Dulbecco's modified Eagle's medium (Gibco) supplemented with 10% fetal bovine serum (Gibco) at 37°C in 5% CO_2_ for luciferase reporter assay [23].

### 2.4. Flow Cytometry Analysis

The isolated PBMCs were resuspended in 100 *μ*l phosphate-buffered saline (PBS), and then 2 *μ*l phycoerythrin-cyanin 5 (PE-Cy5)-labeled antihuman CD3 mAb (Biolegend, California, USA), fluorescein isothiocyanate (FITC)-labeled antihuman CD4 mAb (Biolegend), 2 *μ*l PE-conjugated anti-human CXCR5 mAb (Biolegend), and 2 *μ*l BV-421-conjugated antihuman ICOS mAb (Biolegend) were successively added in the suspended cells. Meanwhile, we set the corresponding isotype control, and then put the samples in a 4°C dark refrigerator for 30 min. After staining, the cells were washed twice and resuspended in 350 *μ*l PBS for FCM using FACS Canto system (Becton, Dickinson, New Jersey, USA). FlowJo 10 (Stanford University, San Francisco, USA) was performed to determine the proportion of cTfh cells and the mean fluorescence intensity (MFI) of ICOS. We defined CD3^+^ CD4^+^ CXCR5^+^ ICOS^+^ cells as cTfh cells.

### 2.5. RNA Extraction and qRT-PCR

Total RNAs were extracted by the RNA-Quick Purification kit (YiShan Biotech, Shanghai, China) from the PBMCs or cultured cells according to the manufacturers' instructions. The complementary DNAs (cDNAs) were synthesized by the ReverTraAca®qPCR RT kit (Toyobo, Osaka, Japan). qRT-PCR was performed with appropriate primers using TB Green Premix Ex Taq Ⅱ (TaKaRa, Osaka, Japan), and the sequences of primers were shown in *Supplementary table 2*. The primer sequences of miR-29a-3p and U6 primers were designed and synthesized by Ribobio Co. (Ribobio, Guangzhou, China) and the patent number was CA201410039162.6. The relative expression of target genes and miR-29a-3p was normalized to the expression of *β*-actin and U6, respectively. Data were analyzed using Applied Biosystems 7500 Manager software (Thermo Fisher Scientific, Waltham, USA).

### 2.6. Sanger Sequencing

Total RNAs were extracted from the PBMCs and synthesized cDNA. The genomic DNA (gDNA) was isolated with DNA Mini Kit (QIAGEN, Stockach, Germany). After the PCR reaction with divergent and convergent primers, the amplified products were subjected to agarose gel electrophoresis. Then, we purified and extracted the DNA from the gel with the PCR product of divergent primers. The DNA samples were sent to Sangon (Sangon Biotech Co., Shanghai, China) for Sanger sequencing [23]. The primers for Sanger sequencing: forward 5′–ACTGCCCAGTGGGAAAAAGA−3′; reverse 5′–TCCATATTGTTGGCAAGCCC−3′.

### 2.7. Ribonuclease R Treatment

To determine the stability of circZNF644, total RNAs (2 *μ*g) were extracted from the PBMCs and treated with 3 U/*μ*g Ribonuclease R (RNase R) (Epicentre Technologies, Wisconsin, USA) for 10 min at 37°C, and finally inactivated for 10 min at 70°C. After treatment with RNase R, we performed qRT-PCR to analyze the relative expression of circZNF644 and ZNF644 mRNA [23].

### 2.8. Nuclear and Cytoplasmic Extraction

According to the manufacturer's instructions, the cytoplasm and nucleus fractions were isolated using the PARIS™RNA purification kit (Thermo Fisher Scientific). Briefly, PBMCs were lysed in a precooled Cell Fractionation Buffer for 10 min. After centrifugation at 4°C 500 g for 5 min, the supernatant was collected as the cytoplasmic fraction and the precipitation as the nuclear fraction. The isolated cytoplasmic and nuclear fractions were used to detect the relative expression of circZNF644. Lamin B1 and GAPDH were used as nuclear internal control and cytoplasmic internal control, respectively [23].

### 2.9. Fluorescence In Situ Hybridization (FISH)

The sequences of circZNF644 and miR-29a-3p probes for FISH were designed and synthesized by GenePharma (GenePharma, Suzhou, China). A FAM-labeled circZNF644 probe (green) and a Cy3-labeled miR-29a-3p probe (red) were shown as follows: circZNF644, 5′–CCATTTAACACATTTAGTCTGAGTGCATAGTCAAGT−3′, miR-29a-3p, 5′–TAACCGATTTCAGATGGTGCTA−3′. The isolated PBMCs were fixed and tested using a FISH kit (GenePharma) according to the manufacturers' instructions. The nucleus was incubated with DAPI for blue. The images were acquired using an OLYMPUS FV1000 confocal microscopy (OLYMPUS, Tokyo, Japan).

### 2.10. RNA Interference (RNAi) and miRNA Transfection

Small interfering RNAs (siRNAs) were designed and synthesized to target-specific circZNF644 (si-circZNF644), and nonspecific scramble siRNA was designed as negative control (si-NC) (RiboBio). MiR-29a-3p inhibitor and negative control (miR-NC) were designed and synthetized by Ribobio Co. (Ribobio). The isolated PBMCs were transfected with si-circZNF644 and si-NC at 100 nM or/and miR-29a-3p inhibitor and miR-NC using Entranster-R transfection reagent (Engreen Biosystem, Co., Ltd., Beijing, China) according to the manufacturers' instructions for 48 hr in the presence of 0.5 *μ*g/ml functional antihuman CD3 mAb plus 2 *μ*g/ml functional antihuman CD28 mAb (Miltenyi Biotec GmbH, Bergisch Gladbach, Germany) before further experiments [23].

### 2.11. Enzyme-Linked Immunosorbent Assay (ELISA)

The cellular supernatant was separated from the transfected PBMCs, and the concentrations of cytokine IL-21 were determined by ELISA according to the manufacturer's instructions (IBL International GmbH, Hamburg, Germany) [22].

### 2.12. Luciferase Reporter Assay

The sequences of wild-type (WT) circZNF644 and mutant circZNF644 were synthesized by Sangon (Sangon Biotech Co.) and cloned into the vector psiCHECK-2 (Promega, Madison, USA). Then, recombinant vectors and miR-29a-3p mimic or miR-NC were cotransfected into HEK293T cells using Lipofectamine 3000 reagent (Thermo Fisher Scientific) as described in our previous study [23].

### 2.13. Statistical Analysis

GraphPad Prism version 8.0.2 software (GraphPad Inc., San Diego, USA) was performed to manage the data. Data were presented as the mean ± standard deviation (SD). A student's unpaired *t*-test was used to evaluate the comparison of two groups when variables passed the normal distribution test. The correlation was determined by Pearson's correlation analysis when the variables passed the normal distribution test, and the correlation between non-normally distributed variables was calculated by Spearman's correlation coefficient [23]. A *p* < 0.05 was considered statistically significant (* ^*∗*^p*  < 0.05, * ^*∗∗*^p*  < 0.01, * ^*∗∗∗*^p*  < 0.001).

## 3. Results

### 3.1. Elevated Expression of CircZNF644 in GD Patients

**Figure 1:**
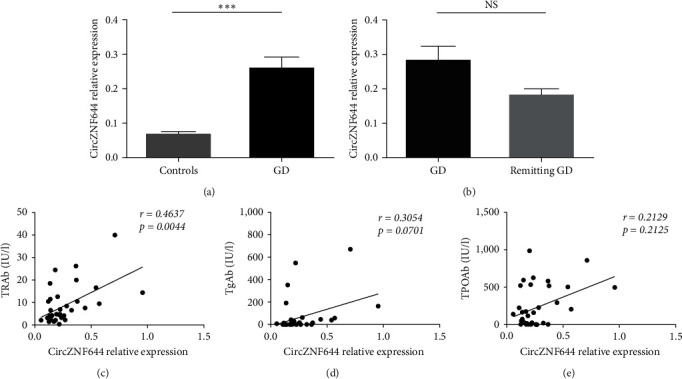
Elevated expression of circZNF644 in GD patients. (a) The expression of circZNF644 was detected by qRT-PCR in the PBMCs of GD patients (*n* = 36) and controls (*n* = 38). (b) qRT-PCR analysis of circZNF644 expression in GD patients (*n* = 28) and remitting GD patients (*n* = 8). The correlations between circZNF644 expression and the serum levels of TRAb, (c) TgAb, (d) TPOAb, and (e) in 36 GD patients. Data are presented as the mean ± SD. Each point represents an individual subject and data are analyzed using Spearman's correlation analysis.  ^*∗∗∗*^*p* < 0.001; NS, no significance.

In order to investigate the transcript levels of circZNF644 in GD patients, we verified the sequencing results (GEO ID: GSE197637) of circZNF644 by expanding the sample size of GD patients and healthy subjects. The data showed that the transcript levels of circZNF644 were increased in the PBMCs of GD patients compared with healthy subjects, which was consistent with the sequencing data (Figure 1(a)). Intriguingly, the transcript levels of circZNF644, which were higher than normal before treatment, decreased after normalization of the thyroid function, but there was no statistical significance between GD patients and remitting GD patients (Figure 1(b)). Next, we investigated the relationship between circZNF644 expression and clinical features. We found that circZNF644 expression was only positively correlated with the serum levels of TRAb (*r* = 0.4637; *p*=0.0044) in GD patients (Figures 1(c), 1(d), and 1(e)). Furthermore, there was also no correlation between the transcript levels of circZNF644 and the levels of thyroid hormones (*Supplementary figure 1*). These results indicated that elevated levels of circZNF644 might participate in the process of GD.

### 3.2. Circular Characteristics of CircZNF644

**Figure 2:**
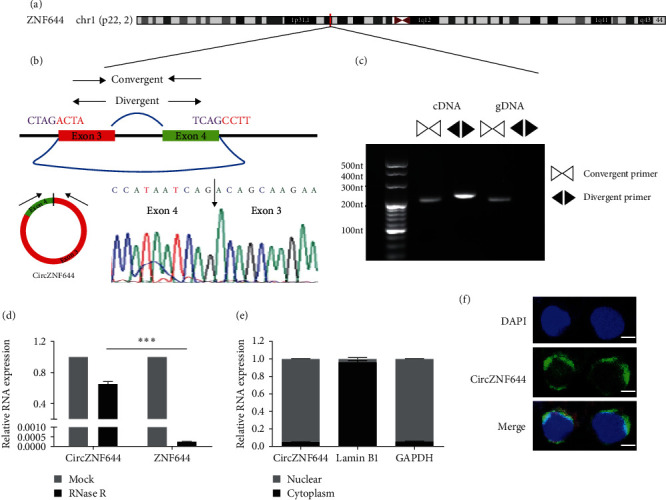
Circular characteristics of circZNF644. (a) Genomic position of circZNF644 on human chromosome. (b) Schematic illustration of circZNF644 formation through the circularization of exons 3 and 4 in ZNF644. The convergent and divergent primers were used to detect circZNF644 and its linear ZNF644 mRNA, respectively. Sanger sequencing following the PCR product of divergent primers confirmed the “head-to-tail” splicing of circZNF644 in the PBMCs. The position of the arrow showed the back-splice junction site. (c) The existence of circZNF644 was validated by RT-PCR products amplified in cDNA and gDNA with convergent and divergent primers, respectively. The RT-PCR product with divergent primer in cDNA shows the circularization of circZNF644. cDNA, complementary DNA; gDNA, genomic DNA. (d) Relative expression of circZNF644 and ZNF644 mRNA after RNase R digestion. (e) Relative expression of circZNF644 in the nuclear and cytoplasm by qPT-PCR. Lamin B1 was performed as the positive control in the nucleus and GAPDH was performed as the positive control in the cytoplasm. (f) Representative fluorescence images of circZNF644 (green) in the PBMCs were obtained by FISH assay. Nuclei were stained with DAPI. Scale bar, 25 *µ*m. Data are presented as the mean ± SD.  ^*∗∗∗*^*p*  < 0.001.

According to the bioinformatics analysis, the circZNF644 transcript, belonged to exonic circRNA, was generated from exon3 and exon4 circularization of the corresponding linear RNA ZNF644 gene located on the chromosome 1p22 (chr1 : 91403041-91406866), which is 3644bp in spliced length (Figure 2(a)). To examine the circular characteristics of circZNF644, we designed convergent and divergent primers for Sanger sequencing and PCR analysis. Sanger sequencing results confirmed the presence of a “head-to-tail” back-splice junction site in circZNF644 (Figure 2(b)). After examined by the PCR product, divergent primer was capable of generating the circular isoform of circZNF644 from cDNA rather than from gDNA (Figure 2(c)). Then, we compared the stability of circZNF644 and the linear ZNF644 transcripts with RNase R treatment (a degrader of linear RNA), and found that circZNF644 could resist RNase R digestion compared with ZNF644 (Figure 2(d)). To further visualize the intracellular expression pattern and localization of circZNF644, we performed the fractionation of nuclear and cytoplasmic experiments and FISH assay in human PBMCs. Notably, circZNF644 was mainly expressed and localized in the cytoplasm (Figures 2(e) and 2(f))). These findings suggested that circZNF644 was a circular and stable transcript, predominantly expressed in cytoplasm.

### 3.3. CircZNF644 Inhibited miR-29a-3p Expression in GD Patients

**Figure 3:**
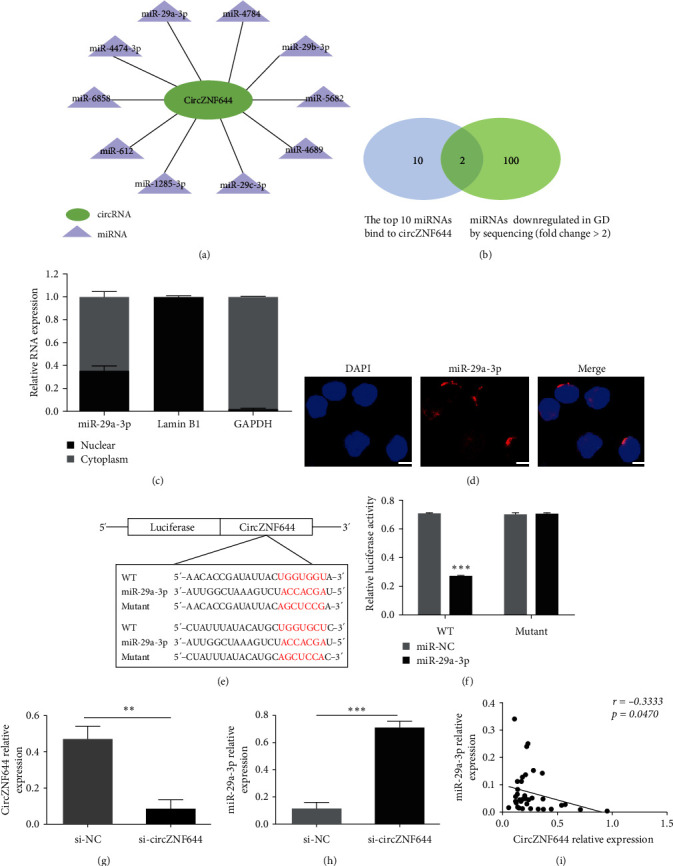
CircZNF644 inhibited miR-29a-3p expression in GD patients. (a) The top 10 miRNAs that bind to circZNF644. (b) Venn diagram revealed the intersection of top 10 miRNAs bound to circZNF644 and downregulated miRNAs from sequencing data (FC > 2). (c) Nucleoplasmic RNA isolation and qRT-PCR for the localization of miR-29a-3p. Lamin B1 was performed as the nuclear positive control, and GAPDH was used as the cytoplasmic positive control. (d) FISH assay was performed to ascertain the localization of miR-29a-3p in the PBMCs. Scale bar, 25 *µ*m. (e) The sequence of circZNF644 was attached to the recombinant eukaryotic expression vector, which was named wild-type (WT), and the mutated sequence of circZNF644, named mutant, was unable to bind miR-29a-3p. (f) A luciferase reporter assay was performed to detect luciferase activity of WT or mutant vectors after cotransfection with miR-29a-3p mimics and miR-NC in HEK293T cells. (g) The relative expression of circZNF644 was detected in the PBMCs after treatment with si-circZNF644 and si-NC. (h) qRT-PCR analysis was performed to investigate miR-29a-3p expression between si-circZNF644 and si-NC group. (i) The correlation analysis was performed to assess the relationship between circZNF644 expression and miR-29a-3p expression in the PBMCs of 36 GD patients. Data are presented as the mean ± SD. Each point represents an individual subject and data are analyzed using Spearman's correlation analysis.  ^*∗∗*^*p*  < 0.01;  ^*∗∗∗*^*p*  < 0.001.

CircRNAs can regulate miRNAs by competitively combining miRNAs through miRNA recognition elements (MRE). Injecting CDR1as expressing plasmids into embryos resulted in the decreased levels of miR-7 in zebrafish. In contrast, knockdown of CDR1as by shRNA and siRNA resulted in increased miR-7 levels and downregulation of miR-7 targets in cancer cell lines [24]. The cytoplasmic localization of circZNF644 suggested that circZNF644 might act as a competing endogenous RNAs (ceRNAs) of miRNAs in GD. To verify the hypothesis, we used TargetScan and miRanda programs to predict the potential miRNAs bind to circZNF644. A total of 727 candidate miRNAs were identified with binding sites on circZNF644. The top 10 related miRNAs were screened in the form of network according to Context^+^ Energy produced by TargetScan and miRanda software (Figure 3(a)). We then examined whether these miRNAs were decreased in the GD sequencing data (GSE183576) (fold change (FC) > 2), and Venn diagram revealed that miR-29a-3p and miR-29b-3p meet the requirement (Figure 3(b)). In the previous validation, only miR-29a-3p expression was notably decreased in the PBMCs of GD patients [22]. Combined with the predictive information and subsequent validation, miR-29a-3p was selected for further study.

Next, we identified the localization of miR-29a-3p and found that miR-29a-3p was also mainly localized in the cytoplasm (Figures 3(a) and 3(b))). Moreover, the costaining result between circZNF644 and miR29a-3p showed that circZNF644 and miR-29a-3p were colocalized in the cytoplasm (*Supplementary figure 1*). Then, we designed the luciferase reporter minigenes vector containing circZNF644 sequence (WT) and mutant vector that is unable to bind miR-29a-3p (Figure 3(c)). The dual-luciferase reporter assay was performed to verify the target relationship, and the data revealed that miR-29a-3p remarkably attenuated the luciferase activity of WT sequence compared with miR-NC, while miR-29a-3p did not affect the luciferase activity of mutant sequence (Figure 3(d)). To further verify the regulatory relationship between circZNF644 and miR-29a-3p, we tried to design siRNA at the back-splicing junction site of circZNF644, and the data showed that knockdown of circZNF644 could increase miR-29a-3p expression (Figures 3(e) and 3(f))). In addition, a significantly inverse correlation between circZNF644 expression and miR-29-3p expression was found in the PBMCs of GD patients (*r* = −0.3333; *p*=0.0470) (Figure 3(g)). These results demonstrated that circZNF644 inhibited miR-29a-3p expression and might influence the biological function of miR-29a-3p in GD patients.

### 3.4. CircZNF644 Regulated ICOS Expression in GD Patients

**Figure 4:**
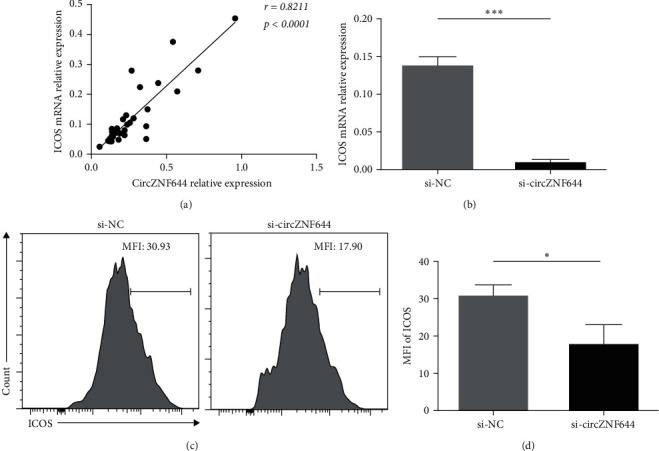
CircZNF644 regulated ICOS expression in GD patients. (a) The correlation between the transcript levels of circZNF644 and the transcript levels of ICOS in 36 GD patients. Each point represents an individual subject, and data are analyzed using Spearman's correlation analysis. CircZNF644-specific siRNA (si-circZNF644) and negative control (si-NC) were designed and transfected into the PBMCs. (b) The transcript levels of ICOS were detected by qRT-PCR after transfection with siRNA into the PBMCs. (c) The schematic diagram of the MFI of ICOS was measured by flow cytometry analysis. (d) The statistical analysis of the MFI of ICOS between si-circZNF644 and si-NC group. Data are presented as the mean ± SD. ^*∗*^*p*  < 0.05;  ^*∗∗∗*^*p*  < 0.001.

Owing to miR-29a-3p could directly inhibit ICOS expression in GD patients [22], we further investigated the relationship between circZNF644 and ICOS in GD patients. A significantly positive correlation between the transcript levels of circZNF644 and the transcript levels of ICOS was shown in the PBMCs of GD patients (*r* = 0.8211; *p* < 0.0001) (Figure 4(a)). To clarify the role of circZNF644 on ICOS expression, we transfected si-circZNF644 and si-NC into human PBMCs, and the results showed that the degradation of circZNF644 inhibited ICOS expression (Figure 4(b)). Consistently, the MFI of ICOS was significantly downregulated in si-circZNF644 group (Figures 4(c) and 4(d))). Taken together, these data suggested that circZNF644 could promote ICOS expression in GD patients.

### 3.5. Regulatory Effect of CircZNF644 on cTfh Cells in GD Patients

**Figure 5:**
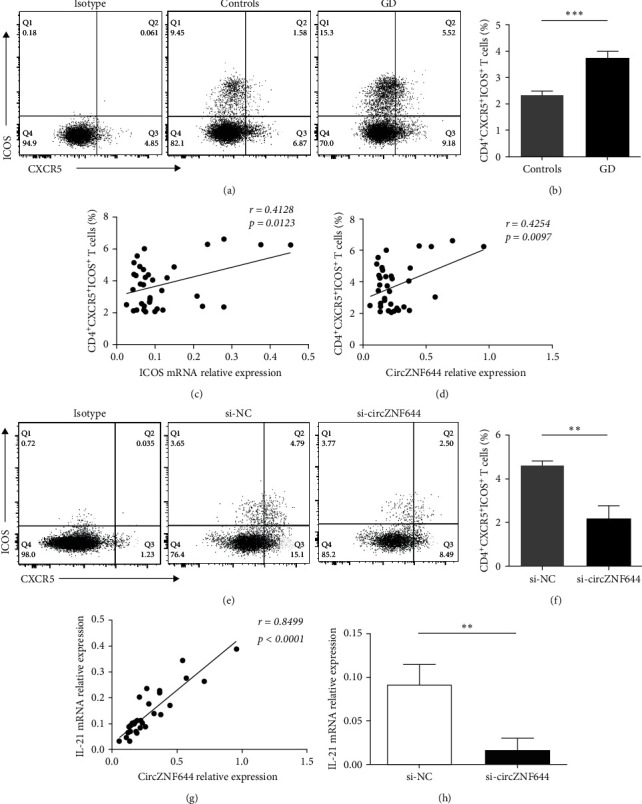
Regulatory effect of circZNF644 on cTfh cells in GD patients. (a) Representative flow cytometry dot plots of cTfh cells in GD patients and controls. The isotype was used to set the quadrant range. (b) The frequency of cTfh cells (CD4^+^CXCR5^+^ICOS^+^ T cells) in the PBMCs of GD patients (*n* = 36) and controls (*n* = 38) was detected by flow cytometry analysis. (c) The correlation between the levels of ICOS mRNA and the proportion of cTfh cells was observed in the PBMCs of 36 GD patients. (d) The correlation between circZNF644 expression and the proportion of cTfh cells in the PBMCs of 36 GD patients. Each data point represents an individual subject and data are analyzed using Pearson's correlation analysis. (e, f) Human PBMCs were transfected with circZNF644 siRNA (si-circZNF644) and negative control (si-NC), and then CXCR5^+^ICOS^+^ cells in CD4^+^ T cells gate were analyzed by flow cytometry analysis. The isotype was used to set the quadrant range. (g) The correlation between circZNF644 expression and IL-21 expression in the PBMCs of 36 GD patients. Each data point represents an individual subject, and data are analyzed using Spearman's correlation analysis. (h) The relative expression of IL-21 mRNA was determined after transfection with si-circZNF644 and si-NC. Data are presented as the mean ± SD.  ^*∗∗*^*p* < 0.01;  ^*∗∗∗*^*p* < 0.001.

**Figure 6:**
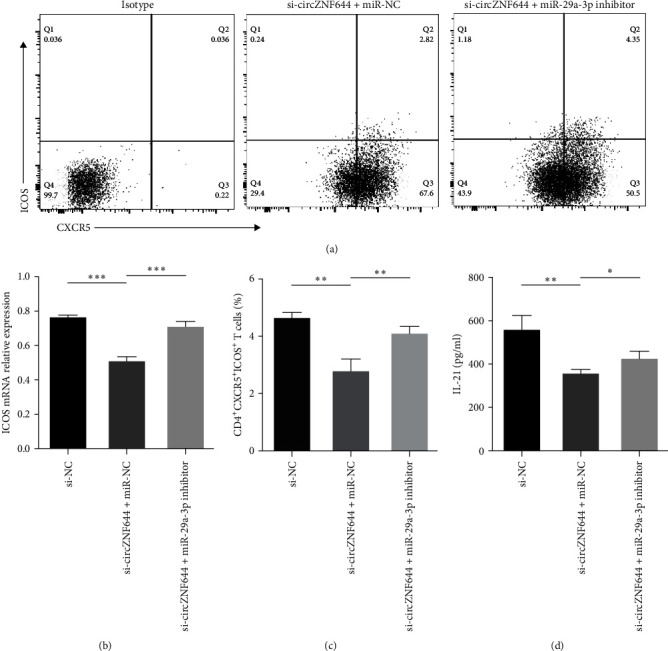
CircZNF644 regulates cTfh cells by influencing the miR-29a-3p/ICOS axis. (a) Representative flow cytometry dot plots of cTfh cells in si-circZNF644 + miR-NC and si-circZNF644 + miR-29a-3p inhibitor groups. The isotype was used to set the quadrant range. (b) The transcript levels of ICOS were determined after transfection with si-NC, si-circZNF644 + miR-NC, and si-circZNF644 + miR-29a-3p inhibitor. (c) The proportion of cTfh cells in si-NC, si-circZNF644 + miR-NC, and si-circZNF644 + miR29a-3p inhibitor groups. (d) The concentrations of IL-21 in cellular supernatant of si-NC, si-circZNF644 + miR-NC, and si-circZNF644 + miR-29a-3p inhibitor groups were determined by ELISA. Data are presented as the mean ± S.D.  ^*∗*^*p* < 0.05;  ^*∗∗*^*p* < 0.01;  ^*∗∗∗*^*p* < 0.001.

To address the hypothesis that circZNF644 contributes to cTfh cells in GD patients, we first gated on CD3^+^CD4^+^CXCR5^+^ICOS^+^ cells to distinguish cTfh cells from the PBMCs (Figure 5(a)). Our data indicated that the proportion of cTfh cells was significantly increased in the PBMCs of GD patients compared with the controls (3.76 ± 1.44 vs. 2.35 ± 0.82) (Figure 5(b)). Meanwhile, we found that ICOS expression was significantly positively correlated with the percentage of cTfh cells in GD patients (*r* = 0.4128; *p*=0.0123) (Figure 5(c)). To assess the relationship between circZNF644 and cTfh cells, our data showed that circZNF644 expression was positively correlated with the proportion of cTfh cells in the PBMCs of GD patients (*r* = 0.4254; *p*=0.0097) (Figure 5(d)). Then, we produced circZNF644 knockdown PBMCs by siRNA transfection. Intriguingly, the proportion of cTfh cells was decreased when circZNF644 was knockdown in vitro (Figures 5(e) and 5(f))). To further confirm their relationship, we investigated the potential role of circZFN644 on the cTfh cell-associated cytokine IL-21, which showed a positive correlation between circZNF644 expression and the levels of IL-21 mRNA in the PBMCs of GD patients (*r* = 0.8499; *p* < 0.0001) (Figure 5(g)). Consistently, Figure 5(h) showed that the transcript levels of IL-21 were significantly decreased after circZNF644 silencing. To further elucidate the role of circZNF644/miR29a-3p/ICOS axis on cTfh cells, we first detected ICOS expression in PBMCs transfected with circZNF644 siRNA alone and both circZNF644 siRNA and miR-29a-3p inhibitor. The data showed that downregulation of circZNF644 alone resulted in decreased levels of ICOS mRNA, whereas miR-29a-3p inhibitor could reverse the inhibiting effect of circZNF644 siRNA on ICOS expression (Figure 6(b)). Moreover, we found that miR-29a-3p inhibitor was also able to reverse the effect of circZNF644 siRNA inhibiting on the proportion of cTfh cells (Figures 6(a) and 6(c)) and the cellular supernatant concentrations of IL-21 (Figure 6(d)). Above all, these results demonstrated that circZNF644 contributed to increased cTfh cells in peripheral blood of GD patients by regulating miR-29a-3p/ICOS axis.

## 4. Discussion

As an organ-specific autoimmune disease, GD patients have high concentrations of autoantibodies, which are produced in the ectopic GCs of the thyroid gland formed by lymphocytes infiltration [25]. While Tfh cells play a central role in the differentiation and autoantibodies production of B cells in GCs [26]. A group of T cells with the characteristics of Tfh cells, named cTfh, is present in human blood, and their frequency and phenotype are often altered in patients with autoimmune diseases [9]. The present data showed that the percentage of cTfh cells was increased in the PBMCs of GD patients, consistent with two previous reports [7, 27]. The association of cTfh cells with thyroid hormones and autoantibody suggests that cTfh cells may participate in GD by influencing the production of autoantibodies by B cells [7]. Hence, the mechanism causing the aberrant accumulation of cTfh cells in GD patients has become one of the important pointcuts to investigate the pathogenesis of GD. Recently, emerging evidence has revealed that a group of noncoding RNAs play pivotal roles in the differentiation and development of Tfh cells [28, 29]. However, little is known regarding cTfh cells regulation by circRNAs. In the present study, we found that circZNF644 emerged as a key checkpoint involved in cTfh cells response in GD patients. Mechanistically, circZNF644 may promote ICOS expression via inhibiting miR-29a-3 p, thus leading to increased cTfh cells.

Circular zinc finger protein 644 (circZNF644, hsa_circ_0114427, chr1: 91403041-91406866) has recently come into the spotlight with the publication of a number of studies in the past few years. It was initially identified in isolated renal tubular tissue from acute kidney injury (AKI) model and involved in the inflammatory progression in AKI's early stage via circZNF644/miR-494/ATF3 axis [30]. Subsequently, Xing et al. [31] found another pathway, that is, circZNF644 aggravated lipopolysaccharide (LPS)-induced HK-2 cells impairment by regulating miR-140-5p/MLKL pathway. Similarly, according to the data from Gong et al. [32], circZNF644 was upregulated in sepsis-induced AKI patients, and knockdown of circZNF644 protected HK-2 cells from LPS-induced injury by altering miR-335-5p/HIPK1 axis. However, circZNF644 has not been reported in autoimmune diseases before our research group reported it. Our previously study screened dysregulated circZNF644 in GD patients using NGS [21]. In this study, our data confirmed that circZNF644 was a stable circRNA overexpressed in the PBMCs of GD patients.

Depending on their localization and specific interactions with various molecules, circRNAs localized in the nucleus can regulate splicing and transcription of nuclear mRNAs, while circRNAs localized in the cytoplasm can modulate stability and translation of cytoplasmic mRNAs or serve as protein decoys, scaffolds, and recruiters or serve as templates for translation in different biological and pathophysiological contexts [33]. The findings of circZNF644 localized in the cytoplasm suggested circZNF644 may serve as a ceRNA to competitively bind miRNAs. Bioinformatics analysis tools were performed to predict the top 10 potential miRNAs that could bind to circZNF644. miR-29a-3p was the only decreased miRNA in GD sequencing data and verification result. Moreover, we also detected the changes of miR-140-5p, miR-494, and miR-335-5p in GD sequencing data owing to the targeting relationship between these miRNAs and circZNF644. The results revealed that miR-335-5p was highly expressed, miR-494 was not detected, and miR-140-5p was low expressed but the fold change was less than 2. Although circZNF644 can regulate these miRNAs in patients with AKI, circZNF644 may play a regulatory role by affecting miR-29a-3p in GD due to the disease-specific condition. Next, miR-29a-3p was first identified as a potential miRNA bind to circZNF644 by prediction programs, and both of them were colocated in the cytoplasm, which provided the condition for their interaction. Second, a significant inverse correlation between elevated circZNF644 levels and reduced miR-29a-3p levels was revealed in the PBMCs of GD patients, suggesting the possibility of circZNF644 regulating miR-29a-3p. Third, miR-29a-3p significantly reduced the luciferase activity of WT-circZNF644, while miR-29a-3p had no effect on the luciferase activity of mutant-circZNF644, suggesting that circZNF644 could directly bind to miR-29a-3p. Lastly, knockdown of circZNF644 augmented miR-29a-3p expression in vitro. The above results also elucidated one of the reasons for the abnormal reduction of miR-29a-3p in GD patients. Our data indicated that circZNF644 could exert its function by inhibiting miR-29a-3p in GD patients.

So far, miRNA, as a small endogenous noncoding RNA, also needs to exert biological functions by regulating mRNAs [34]. Our previous data showed that ICOS was a functional target of miR-29a-3p [22]. In this study, there was a strong relationship between the transcript levels of circZNF644 and ICOS expression in the PBMCs of GD patients. In addition, knockdown of circZNF644 suppressed ICOS expression at both transcriptional and translational levels in vitro. Intriguingly, this inhibition phenomenon could be reversed by miR-29a-3p inhibitor. However, the decreased MFI levels of ICOS were not as pronounced as the reduction in the transcript levels of ICOS, which may be due to the difference in the time and site between transcription and translation. It is possible that mRNA has been completely degraded and the protein has not reached the degradation trough. These findings meant that miR-29a-3p might be serve as a bridge between circZNF644 and ICOS. ICOS was first reported to be highly expressed on GC CD4^+^ T cells [35], and then it was found to express on the surface of Tfh cells [36]. ICOS-mediated PI3 kinase (PI3K) activation is dispensable for Tfh cells differentiation and is also critical for the induction of the key Tfh cytokine, IL-21 [37]. A study reported that ICOS^high^ cTfh cells produced higher levels of IL-21 and IgG antibodies than ICOS^low^ cTfh cells [38]. The present data showed that the transcript levels of ICOS were positively correlated with the proportion of cTfh cells in GD patients. Combined with the strong relationship between ICOS expression and IL-21 expression in GD [22], we speculated that circZNF644 might contribute to increased cTfh cells in GD patients by regulating ICOS, which was confirmed by a series of experiments. These findings demonstrated that circZNF644 facilitated ICOS expression via regulating miR-29a-3p, and it functioned to cTfh cells may act as a ceRNA for miR-29a-3p. But much detail needs to be further investigated.

Clinically, the signs and symptoms of GD are shared by other forms of hyperthyroidism [39]. However, their pathogenic mechanisms are different. Graves' hyperthyroidism is caused by TRAb, which bind to thyroid follicular cells to activate TSH receptor [40]. In the present study, circZNF644 expression was significantly positively correlated with the serum levels of TRAb in GD patients, which was consistent with the relationship between cTfh cells and laboratory parameters [22], suggesting that circZNF644 might affect the production of TRAb by Tfh-B cells pathway in GD. Meanwhile, we found that circZNF644 was moderately associated with thyroid hormone levels and TgAb/TPOAb levels. One possible explanation for this phenomenon is that the cohort of GD patients is insufficient. Another possible explanation is that although TgAb and TPOAb are found in part GD patients, TRAb is the most relevant autoantibody to GD [41]. The present study has certain limitations. On the one hand, it was a single-center study with a limited sample size, and larger cohorts of GD patients should be enrolled in the future study. On the other hand, we only investigated the mechanisms of circZNF644 on cTfh cells in vitro. The detailed mechanisms of circZNF644 should be further investigated in the mice model of GD.

## 5. Conclusion

Collectively, our group found a stable elevated expression of circZNF644 in peripheral blood of GD patients, which might contribute to the pathogenic role of cTfh cells response in the process of GD.

## Figures and Tables

**Table 1:** Baseline clinical characteristics of study participants.

Variables	GD patients	Controls	Range	*p*
Number	36	38	—	—
Gender (M/F)	9/27	13/25	—	0.386
Age (year)	40 ± 10	40 ± 10	—	0.817
FT3 (pmol/l)	10.83 ± 10.43	5.24 ± 0.96	3.28–6.47	0.002
FT4 (pmol/l)	20.99 ± 17.50	11.59 ± 1.23	7.64–16.03	0.002
TSH (uIU/ml)	0.79 ± 1.46	2.14 ± 1.08	0.56–5.91	<0.001
TgAb (IU/ml)	63.61 ± 151.40	0.39 ± 0.49	0–4	0.012
TPOAb (IU/ml)	228.85 ± 270.74	0.72 ± 0.93	0–9	<0.001
TRAb (IU/l)	8.48 ± 8.54	<0.80	0–1.75	<0.001

Data correspond to the arithmetic mean ± SD and were compared using unpaired *t*-tests or Chi-square test. M, male; F, female. The data exceed the instrument detection range as follows: TRAb < 0.80 IU/l.

## Data Availability

The sequencing datasets of circRNAs can be found in the GEO/GSE197637. The data that support the findings of this study are available from the corresponding author upon reasonable request.

## Additional Points

*Abbreviated Title*. CircZNF644 promotes cTfh cells in GD.

## Supplementary Materials

Supplementary 1 Figure 1: the correlations between CircZNF644 expression and the serum levels of thyroid hormones, including FT3 (a), FT4 (b) and TSH (c) in 36 GD patients. Each data point represents an individual subject. Data are analyzed using Spearman's correlation analysis. Figure 2: the colocalization of CircZNF644 and miR-29a-3p in the PBMCs by FISH. Scale bar, 25 *µ*m.

Supplementary 2 Table 1: primer sequences of the genes in the study.
