# Supplementary material for: Circular RNA CircZNF644 Facilitates Circulating Follicular Helper T Cells Response in Patients with Graves' Disease
Source: J Immunol Res. 2024 Jun 27;2024:9527268. doi: 10.1155/2024/9527268 (PMC11223900; doi:10.1155/2024/9527268)
Supplement: Supplementary 1 — Figure 1: the correlations between CircZNF644 expression and the serum levels of thyroid hormones, including FT3 (a), FT4 (b) and TSH (c) in 36 GD patients. Each data point represents an individual subject. Data are analyzed using Spearman's correlation analysis. Figure 2: the colocalization of CircZNF644 and miR-29a-3p in the PBMCs by FISH. Scale bar, 25 µm. [file 9527268.f1.docx]

F_IGURE_ S1: The correlations between circZNF644 expression and the serum levels of thyroid hormones, including FT3 (a), FT4 (b) and TSH (c) in 36 GD patients. Each data point represents an individual subject. Data are analyzed using Spearman’s correlation analysis.

F_IGURE_ S2: The colocalization of circZNF644 and miR-29a-3p in the PBMCs by FISH. Scale bar, 25 µm.
