# Supplementary material for: Circular RNA CircZNF644 Facilitates Circulating Follicular Helper T Cells Response in Patients with Graves' Disease
Source: J Immunol Res. 2024 Jun 27;2024:9527268. doi: 10.1155/2024/9527268 (PMC11223900; doi:10.1155/2024/9527268)
Supplement: Supplementary 2 — Table 1: primer sequences of the genes in the study. [file 9527268.f2.docx]

T_ABLE_ S1. Primer sequences of the genes in the study

| PRIMER ID | NUCLEOTIDE | SEQUENCE (5'-3') | |
| --- | --- | --- | --- |
| circZNF644 | NM_201269 | forward | ACCATCCTCACCCACCTCTACTTG |
|  |  | reverse | ACCTCTGCTTCAGTTGGTTGTGAC |
| ICOS | NM_012092.4 | forward | CAGGAGAAATCAATGGTTCTGCC |
|  |  | reverse | CCTTTTGTCTTAGTGAGATCGCA |
| IL-21 | NM_001207006.3 | forward | CAAGGTCAAGATCGCCAC |
|  |  | reverse | AGGGACCAAGTCATTCACATAA |
| β-actin | NM_001101.5 | forward | CACGAAACTACCTTCAAC |
|  |  | reverse | CATACTCCTGCTTGCTGATC |
| ZNF644 | NM_016620.4 | forward | AGCAAAGTGGAAGGTCAGGA |
|  |  | reverse | TGGGCAGTAAGGACACGATT |
| Lamin B1 | NM_001198557.2 | forward | GGAAGTGAGCGGCATCAAGGAG |
|  |  | reverse | ACTGACCTGGCACGGAGATCC |
| GAPDH | NM_00189745.3 | forward | ACAACTTTGGTATCGTGGAAGG |
|  |  | reverse | GCCATCACGCCACAGTTTC |
